# Prediction of enhancer–promoter interactions using the cross-cell type information and domain adversarial neural network

**DOI:** 10.1186/s12859-020-03844-4

**Published:** 2020-11-07

**Authors:** Fang Jing, Shao-Wu Zhang, Shihua Zhang

**Affiliations:** 1 Key Laboratory of Information Fusion Technology of Ministry of Education, School of Automation, Northwestern Polytechnical University, 127 West Youyi Road, Xi’an, 710072 Shaanxi China; 2 NCMIS, CEMS, RCSDS, Academy of Mathematics and Systems Science, Chinese Academy of Sciences, 55 Zhongguancun East Road, Beijing, 10090 China; 3 School of Mathematical Sciences, University of Chinese Academy of Sciences, Beijing, 100049 China; 4 Center for Excellence in Animal Evolution and Genetics, Chinese Academy of Sciences, Kunming, 650223 China

**Keywords:** Enhancer–promoter interactions, Cell line, Convolutional neural network, Transfer learning, Gradient reversal layer

## Abstract

**Background:**

Enhancer–promoter interactions (EPIs) play key roles in transcriptional regulation and disease progression. Although several computational methods have been developed to predict such interactions, their performances are not satisfactory when training and testing data from different cell lines. Currently, it is still unclear what extent a across cell line prediction can be made based on sequence-level information.

**Results:**

In this work, we present a novel Sequence-based method (called SEPT) to predict the enhancer–promoter interactions in new cell line by using the cross-cell information and Transfer learning. SEPT first learns the features of enhancer and promoter from DNA sequences with convolutional neural network (CNN), then designing the gradient reversal layer of transfer learning to reduce the cell line specific features meanwhile retaining the features associated with EPIs. When the locations of enhancers and promoters are provided in new cell line, SEPT can successfully recognize EPIs in this new cell line based on labeled data of other cell lines. The experiment results show that SEPT can effectively learn the latent import EPIs-related features between cell lines and achieves the best prediction performance in terms of AUC (the area under the receiver operating curves).

**Conclusions:**

SEPT is an effective method for predicting the EPIs in new cell line. Domain adversarial architecture of transfer learning used in SEPT can learn the latent EPIs shared features among cell lines from all other existing labeled data. It can be expected that SEPT will be of interest to researchers concerned with biological interaction prediction.

## Background

The enhancer–promoter interactions (EPIs) play a critical role in gene regulation in eukaryotes. In genetics, a promoter is a region of DNA sequence upstream of a particular gene [1]. The length of a promoter is probably hundreds to thousands of base pairs [2]. Its function aims to initiate gene transcription of a particular gene. While an enhancer is also an important transcriptional regulatory short DNA fragments that further activate the level of transcription of its target genes by contacting close physical proximity to the promoters in the three-dimensional (3D) nuclear space [3]. Hundreds of thousands of enhancers have been estimated to be contained in the human genome. Normally, a promoter is under the control of multiple enhancers, and multiple promoters can be regulated by a single enhancer. Additionally, the distances between interacting enhancer and promoter pairs have varied widely, varying from kilobases to millions of base-pairs because of the chromatin folding in the 3D space [4–8]. Moreover, as more and more studies reported that enhancer sequence variations are associated with serious human diseases [9–12]. Thus, the importance of EPIs for gene expression is matter-of-course.

Over the past decade, many high-throughput experimental approaches, such as chromosome conformation capture-based (3C) [13] and its variants of Hi-C [14] and ChIA- PET [1], have been developed to study the chromatin interactions. Although Hi-C and ChIA-PET could measure the whole genome DNA-DNA interactions, the genomic resolutions are often low, varying from few kilobases to tens of thousands bases [15–17]. In order to study EPIs, very high (< 10 kb) resolution data is needed. All these experimental approaches are technically challenging, time-consuming and have high false-negative rate. What is more, the EPIs vary across different cellular conditions and tissues [18]. While the number of 3D chromatin interaction experiments continue to increase, it is still not possible to perform chromatin interaction experiments for all types of cell and tissues. Therefore, computational approaches are urgently desired to complement experimental protocols.

Due to the limitations of experimental approaches, the number of available experimental data of EPIs is still limited. Several computational methods have been developed to predict EPIs of the genome. Depending on the type of the input data, computational methods can mainly be divided into two categories: DNA sequence-based methods and epigenomic data-based methods. For DNA sequence-based methods, PEP [19] and EP2vec [20] took advantage of natural language processing to learn the feature representation of DNA sequences, and SPEID [21] used convolutional neural network to learn the feature representation of DNA sequences. Recently, Zhuang et al.[22] introduced a novel method to improve the prediction performance of EPIs by using the existing labeled data to pretrain a convolutional neural network (CNN), then adopting the training data from the cell line of interest to continue to train the CNN. Above these methods can accurately predict cell line-specific EPIs from genomic sequence, but they work well only when the training and testing data are from the same cell lines. For the epigenomic data-based methods, IM-PET [23], RIPPLE [24], TargetFinder [2], EpiTensor [25], and JEME [26] used many one-dimensional (1D) local chromatin states including but not limited to transcription factors (TFs) binding, histone modifications and chromatin accessibility signatures to predict EPIs. Though achieving acceptable performance, these models rely on labeled training data from the same cell line as the test data, which limits their usefulness for new cell lines. Generally speaking, high-resolution chromatin interactions experimental data are hard to get, and the cell-specific models have no acceptable generalization due to the specificity of EPIs. Therefore, how to predict the EPIs of new cell line is an urgent problem. And the very intuitive idea is that to train a generic model which learns shared features between different cell lines, and then to predict the EPIs of the new cell line. However, this idea has its drawbacks. The feature distribution learned from the general model is different from that of a particular cell line that we care about. If the distribution of features learned from multiple cell lines is as similar as possible to the distribution of features in the cell line that we care about, then we can make more accurate predictions. Therefore, it is important to develop an effective method to make feature distribution as consistent as possible for transfer knowledge from other cell lines to a specific cell line that we care about.

It is well known that transfer learning (TL), an important branch of machine learning, is widely concerned in image recognition [27] and natural language processing [28], which focuses on the application of knowledge transfer onto new problems. Inspired by the work of [29], which suggested an adversarial neural network by gradient reversal layer (GRL) to fit the feature distribution of source domain and target domain for recognizing handwritten numbers, we proposed a novel method of SEPT to predict EPIs in new cell line. There are no EPI labels information and only has the locations of enhancers and promoters in a particular cell line. The enhancer–promoter pairs of a particular cell line with no labels are defined as target domain, and labeled enhancer–promoter pairs from other cell lines are defined as source domain. Our goal is to learn the features from the source domain, and further reduce the data distribution differences between the target domain and the source domain through adversarial learning. First, we used convolution and long short-term memory (LSTM) layers to learn the features of EP pairs from the enhancer and promoter sequences. Then, adversarial neural network with GRL was used to reduce domain-specific features. GRL could reverse the direction of the gradient by multiplying the gradient with a negative constraints value. Finally, the trained model learns the EPI-related features from the source domain to predict the EPIs of the target domain.

SEPT is of great significance for three points. (i) It could be used as an alternative to the experimental methods, helping other tasks such as identifying mechanisms of SNPs from genome-wide association studies (GWAS) [30]. (ii) It could reveal to what extent EPIs of one cell line could be recognized by data from other cell lines. (iii) It could improve our understanding of gene regulation and disease progression. To this end, we adopted two strategies: one is to combine training data of different cell lines into one unit, and the other is to design a model of SEPT with transfer learning [31] to transfer the informative features from the combined unit to a new cell line. The experimental results show that SEPT has better performance than several other methods. Model architecture analysis shows that long short-term memory (LSTM) layer, and GRL layer are important for across cell line EPIs prediction. Convolution kernels analysis shows that SEPT can effectively capture sequence features that determine EPIs.

## Results

### Comparison with other existing state-of-the-art methods

**Fig. 1:**
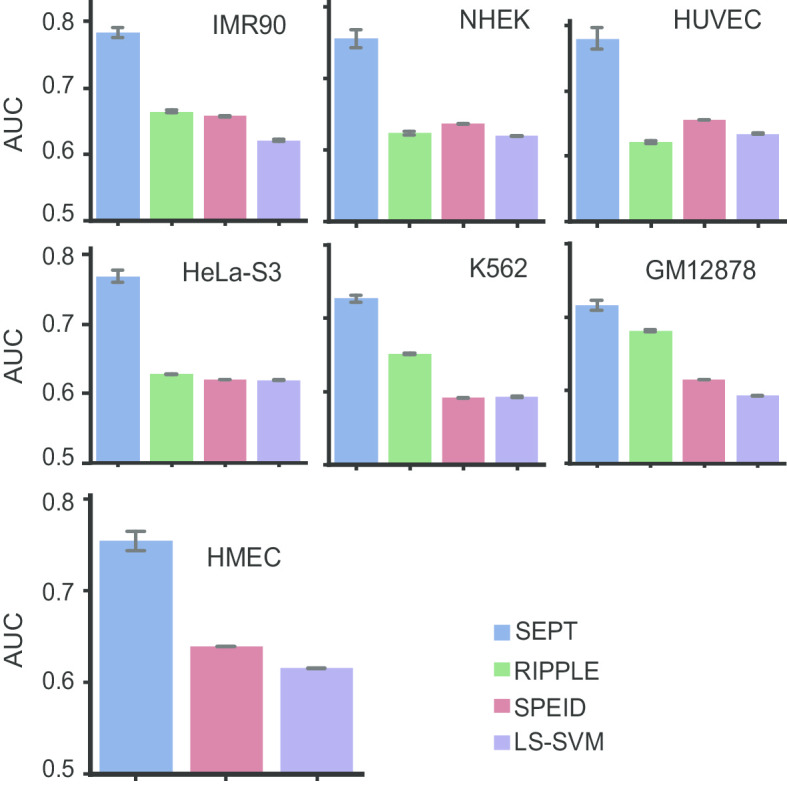
Average AUC of SEPT, RIPPLE, SPEID and LS-SVM on seven test cell lines

We first compared SEPT with other state-of-the-art methods of LS-SVM [32], SPEID [21] and RIPPLE [24]. SPEID and LS-SVM are the two sequence-based methods for predicting the DNA regulatory elements. LS-SVM [32] widely used the *k*-mer features, and did not take into account the interactions of high-order features. Because the LS-SVM [32] can only take one DNA sequence as an input sample, so we concatenated the sequences of the enhancer and promoter to train and test LS-SVM. SPEID [21] used deep learning to capture sequence features for predicting the cross-cell-line EPIs, while it lacks the ability to contain cell-specific information. RIPPLE [24] is a supervised model based on epigenomic data from ChIP-seq and DNase-seq experiments, and it only used five cell lines data to train the model, due to HMEC cell line lack of the epigenomic data. Our SEPT method simultaneously considers the source and target domain sequence information. For each test cell line, data from the other six cell lines were merged into a training data set to train SPEID [21], LS-SVM [32] and SEPT. The average results of SEPT, RIPPLE, SPEID and LS-SVM on seven test cell lines are shown in Fig. 1, from which we can see that SEPT has the highest average AUC values on seven cell lines than RIPPLE, SPEID and LS-SVM. SEPT achieves 0.72, 0.76, 0.78, 0.77, 0.78, 0.73, and 0.76 for GM12878, HMEC, HUVEC, HeLa-S3, IMR90, K562, NHEK cell line, respectively. SEPT also has the highest average AUPR and F1-score values on seven cell lines than RIPPLE, SPEID and LS-SVM (Additional file 1: Tables S1-S2). These results demonstrate that our SEPT can effectively predict the enhancer–promoter interactions.

### Influence of different neural network architectures in feature learning phase

We designed a series of computational network architectures in feature learning phase (Additional file 1: Table S3) to investigate the impact of network structure. The first network architecture (namely BASE) includes only one convolutional layer in feature learning phase (Additional file 1: Figure S1(a)). The second network architecture (namely BASE + LSTM) includes one convolutional layer and one LSTM layer in feature learning phase (Additional file 1: Figure S1(b)). The third network architecture (namely BASE + FC) include one convolutional layer and one full connection layer in feature learning phase (Additional file 1: Figure S1(c)). SEPT includes two convolutional layers and one LSTM layer in feature learning phase (Additional file 1: Figure S1(d)). The grid search strategy was used to optimize the hyperparameters of four models in this work.

**Fig. 2:**
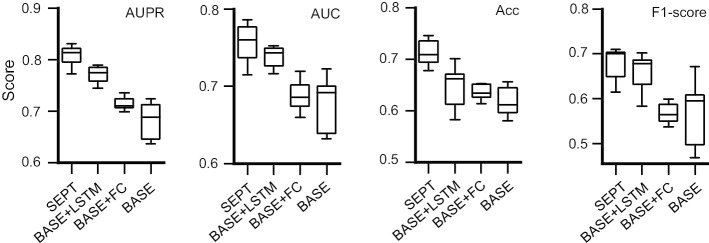
Average results of the BASE, BASE + LSTM, BASE + FC and SEPT on seven test cell lines with the optimal hyperparameters

The average results of BASE, BASE + LSTM, BASE + FC and SEPT on seven test cell lines are shown in Fig. 2, from which we can see that adding the full connection layer (BASE + FC) and the LSTM layer (BASE + LSTM) in BASE model can improve the predictive performance of EPIs. Especially, the results of BASE + LSTM is better than that of BASE + FC, which indicates that the long-range dependency of DNA features such as motifs and other features can be captured by the LSTM layer. SEPT shows the best performance than BASE + LSTM, BASE + FC and BASE models, indicating that the first CNN layer learns the individual patterns in the sequences and the second CNN layer learns the high-order interactions between patterns. The high- order interactions may be commonalities among different cell lines, and deep neural network architectures can extract the high-order EPI features from DNA sequences.

### Influence of domain adversarial operation

To validate the effectiveness of domain adversarial operation, we constructed the model of SEP, which has no the domain adversarial network architecture compared with SEPT. Since SEP has no the domain adversarial operation, the data of target domain cannot be utilized in SEP. That is, SEP just used the data of source domain in the training phase. Table 1 shows the average AUC results of SEPT and SEP by training model on one cell line data and test on another cell line data in running 10 times, from which we can see that AUC values of SEPT for each test cell line are higher than that of SEP. The average AUPR and accuracy values of SEPT for each test cell line are also higher than that of SEP (Additional file 1: Table S4-S5). These results indicate that domain adversarial operation can effectively improve the predictive performance of EPIs in new cell lines, and SEPT makes use of the EPIs information in other cell lines for recognizing the EPIs in new cell lines.

**Table 1:** Average AUC values of SEPT and SEP by training model on one cell line data and test on another cell line data in running 10 times

Model	Test train	GM12878	HMEC	HUVEC	HeLa-S3	IMR90	K562	NHEK
SEPT	GM12878	*	0.63	0.68	0.66	0.67	0.62	0.62
	HMEC	0.59	*	0.57	0.61	0.67	0.56	0.59
	HUVEC	0.62	0.62	*	0.66	0.63	0.58	0.64
	HeLa-S3	0.61	0.62	0.69	*	0.66	0.63	0.66
	IMR90	0.58	0.65	0.62	0.59	*	0.56	0.62
	K562	0.64	0.61	0.66	0.66	0.62	*	0.64
	NHEK	0.62	0.63	0.66	0.67	0.66	0.59	*
SEP	GM12878	*	0.57	0.62	0.59	0.57	0.55	0.55
	HMEC	0.51	*	0.53	0.54	0.60	0.50	0.55
	HUVEC	0.59	0.55	*	0.58	0.57	0.56	0.55
	HeLa-S3	0.56	0.56	0.60	*	0.58	0.54	0.58
	IMR90	0.53	0.61	0.56	0.55	*	0.51	0.57
	K562	0.56	0.53	0.54	0.53	0.53	*	0.53
	NHEK	0.53	0.57	0.56	0.55	0.56	0.50	*

We also investigated the effectiveness of domain adversarial operation by training model on the six cell lines data and test on one other cell line data. The average AUC values of SEP and SEPT in running 10 times are shown in Table 2, from which we can see that average AUC of SEPT is higher 6% ~ 9% than that of SEP on seven test cell lines. The average AUPR, F1-score and accuracy values of SEP and SEPT in running 10 times are shown in Additional file 1: Table S6. These results show that the domain adversarial operation is also effective when using more cell lines data as the training data.

**Table 2:** Average AUC values of SEPT and SEP by training on the six cell lines data and test on one other cell line data in running 10 times

Test cell line	Cell line(s) of source domain	Model	AUC
GM12878	HMEC, HUVEC, HeLa-S3, IMR90, K562, NHEK	SEP	0.65
		SEPT	0.72
HMEC	GM12878, HUVEC, HeLa-S3, IMR90, K562, NHEK	SEP	0.68
		SEPT	0.76
HUVEC	GM12878, HMEC, HeLa-S3, IMR90, K562, NHEK	SEP	0.69
		SEPT	0.78
HeLa-S3	GM12878, HMEC, HUVEC, IMR90, K562, NHEK	SEP	0.69
		SEPT	0.77
IMR90	GM12878, HMEC, HUVEC, HeLa-S3, K562, NHEK	SEP	0.72
		SEPT	0.78
K562	GM12878, HMEC, HUVEC, HeLa-S3, IMR90, NHEK	SEP	0.65
		SEPT	0.73
NHEK	GM12878, HMEC, HUVEC, HeLa-S3, IMR90, K562	SEP	0.69
		SEPT	0.76

### Results comparison of using different number of cell lines as the source domain data

**Fig. 3:**
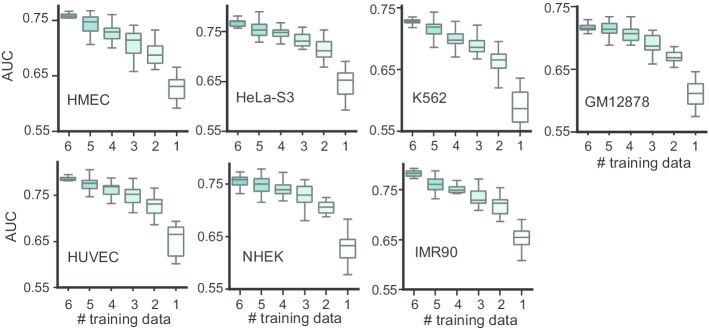
AUC boxplots of different number of cell lines as the source domain in SEPT. The horizontal axis represents the number of cell lines in the source domain

To investigate the influence of different cell line number used in the source domain, we used different number of cell lines as the source domain data to predict the EPIs with SEPT. For selecting 1, 2, 3, 4, 5, 6 cell lines as the source domain data, each test cell line has 6, 15, 20, 15, 6, 1 results, respectively. The AUC results of using different number of cell lines as the source domain data to train SEPT are shown in Fig. 3. Each boxplot in Fig. 3 represents the results of one test cell line across all combinations of source domain cell lines. To make the results more reliable, we repeated 10 times, that is, the AUC value of each test cell line is the mean of 10 running results. From Fig. 3, we can see that more cell lines as the source domain can achieves higher AUC than less cell lines as the source domain. Especially, when 6 cell lines are mixed as the source domain data to train the model, SEPT achieves the highest AUC values for every test cell line. With the increase of the cell lines number in source domain, AUC value is gradually larger. In addition, for different test cell line, the combination of appropriate cell lines as the source data can improve the performance of SEPT. These results show that using more existing labeled data of EPIs is helpful the prediction of EPIs in new cell line.

### Motifs identified by SEPT

As to investigate the motifs, we identified sequence features for each model by comparing patterns of the convolutional kernels in the first layer with sequence motifs from the database HOCOMOCO Human v11. We reconstructed the output of the first convolutional layer for each input sample sequence, then extracted the subsequence that best match each kernel to compute the position frequency matrix (PFM) from the aligned sub sequences for each kernel. The motif comparison tool of Tomtom [33] was used to match these PFMs to known TF motifs. After obtaining the sequence motifs of the two models, we defined a formula to measure the relative importance of motifs. The formula will consider the occurrence times of the same motif in both models and the ranks of the occurrence times of the motif in SEPT. If a motif appears more in SEPT and less in SEP, then the greater the effect of the motif in SEPT, the higher its relative importance score will be. The formula is defined as: $\mathrm{Relative importance}=\left(1-{N}_{wo}/{N}_{w}\right)/{Rank}_{w}$. Here ${N}_{w}$ and ${N}_{wo}$ are the occurrence times of motif learned from SEPT and SEP, respectively; ${Rank}_{w}$ is the rank (in descending order) of the motif in the motif set of SEPT according to the occurrence times. The closer the value is to 1, the more important the motif is in SEPT. To avoid contingency, this process was repeated five times.

We found a set of potentially important transcription factor binding motifs by the transfer model. For each test cell line, top five import motifs involved in EPIs are show in Additional file 1: Table S7. For different test cell lines, the models learned common important TF motifs such as ZNF563, THAP1, RXRA, and SP3, which are involved in many important processes, such as the transcriptional regulation and cell-cycle regulation. Interestingly, we found many of potentially important transcription factors that are associated with the corresponding cell lines (Table 3). For instance, MAFB motif learned by SEPT in the K562 cell line is associated with regulating lineage-specific hematopoiesis. This is consistent with the fact that K562 belong to a blood related cell line. NR4A1 motif learned by SEPT in GM12878 cell line was reported to play a role in the vascular response to injury, while ZNF341 motif learned by both SEPT and SEP in GM12878 cell line was reported to involve in the regulation of immune homeostasis. It is consistent with the fact that GM12878 is lymphoblastoid related cell lines. We also provided other motifs identified by both SEPT and SPEID (Additional file 1: Table S8). These results show that our SEPT can learn important motifs, and these motifs are relevant to enhancer–promoter loops of a novel cell line.

**Table 3:**
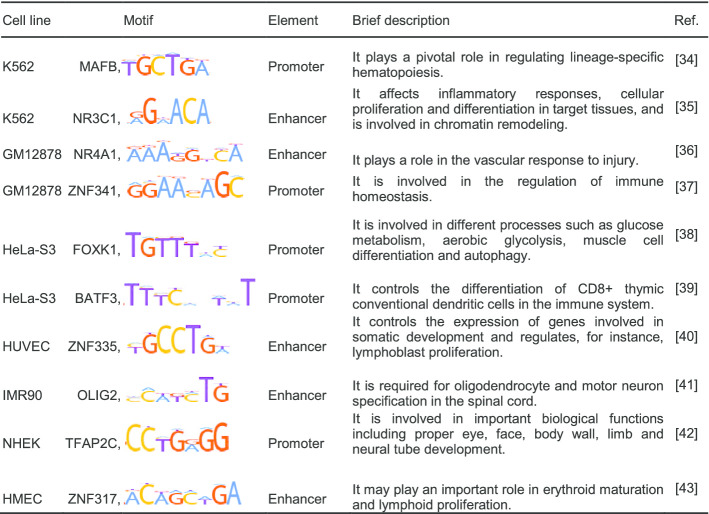
Examples of specific motifs identified by the first convolution layer of SEPT

Motif logos are downloaded from public database HOCOMOCO Human v11.

## Discussions

One important factor in SEPT is how to aggregate the labeled data of different cell lines into a source domain. We investigated the aggregation of different number of cell lines, and found that aggregating all available labeled data of cell lines as the source domain for training model can yield better performance than aggregating partial cell lines data as the source domain. In addition, there is redundancy among different cell lines. Redundancy not only slows down model training, but also damages prediction performance. Thus, how to aggregate different cell lines as the source domain data is important.

How to integrate other features such as histone modifications, chromatin accessibility and DNA shape into one model for predicting EPIs is also important factors. Although we only use sequence information in this work, the results of are still better than other methods which integrate the sequence and epigenomic information. Thus, if more information related to enhancers and promoters is integrated into our SEPT, it is hope that SEPT can significantly improve the performance of EPIs prediction.

Although SEPT can predict the potential EPIs in new cell line, it needs to provide the location of the enhancers and promoters in advance. Therefore, it is need to develop new methods for identifying the EPIs in specific cell line without enhancer and promoter location information.

## Conclusions

Although some deep learning methods have been developed to predict EPIs within the same cell, they cannot get a good performance for predicting EPIs in the unlabeled cell lines, due to lack of understanding of the interested cell lines. In this work, we proposed a transfer learning model to predict EPIs in interested (or “new”) cell lines. To better leverage the existing EPIs knowledge, we adopt the adversarial learning mechanism to learn useful features in the existing labeled cell lines and interesting unlabeled cell lines. Experiment results with the domain adversarial operation indicate that it is helpful to predict EPIs in new cell lines. We expect that the model could learn informative features cross domains and reveal some commonalities (common TFs) between source and target domains. By learning commonalities between the source and target domains, SEPT outperforms other state-of-the-art methods for predicting EPIs in new cell lines.

Although SEPT can effectively predict EPIs in specific cell lines from enhancer and promoter sequences, it can be further improved by considering the following factors. (1) SEPT just uses the sequence information of enhancers and promoters. Integrating other experimental data such as core histone modification ChIP-seq data or DNase-seq data can improve the performance of SEPT. (2) Each cell line is treated equally in the source domain, but the contribution of different cell lines should be different for the test cell line. Determining which cell lines should be used as training data is still needed to be explored, as more and more labeled data will become available. (3) Some EPIs maybe have the cell line specificity, while others are universal across many cell lines. Thus, different samples within the same cell line should have different contribution for cross-cell prediction. Assigning a proper weight to each sample can also improve the performance of SEPT. It can be expected that SEPT can be helpful in other biological interactions prediction scenarios [44, 45], such as the detection lncRNA-miRNA interactions.

## Material and methods

### Data and preprocessing

We used the same Hi-C data as [2], and downloaded the Hi-C data of seven cell lines of K562 (mesoderm-lineage cells from a patient with leukemia), GM12878 (lymphoblastoid cells), HeLa-S3 (ectoderm-lineage cells from a patient with cervical cancer), HUVEC (umbilical vein endothelial cells), IMR90 (fetal lung fibroblasts), NHEK (epidermal keratinocytes) and HMEC (mammary epithelial cells) from Gene Expression Omnibus (GEO) GSE63525. The human reference genome hg19 was used to define the genomic locations. Promoters and activate enhancers in the first four cell lines were identified using segmentation-based annotations from both ENCODE Segway [46] and ChromHMM of Roadmap Epigenomics [47], only ChromHMM annotations were used in the other cell lines. Then, RNA-seq data from ENCODE were used to select activate promoters according to the rule of their mean FPKM > 0.3 with irreproducible discovery rate < 0.1 for each cell line. The genome-wide Hi-C measurements were used to annotate all enhancer–promoter pairs as interacting or non-interacting in each cell type. For each enhancer–promoter pair, the distance between promoter and enhancer of the pair is more than 10 kb and less than 2 Mb [2]. To exclude the effect of distance on determining EPIs, interacting enhancer–promoter pairs were assigned to one bin (the total bin number is 5) based on quantile discretization of the distance between the enhancer and promoter. Random non-interacting pairs of active enhancers and promoters were assigned to their corresponding bin and then subsampled as the same number of positive samples within each bin. The subsampled non-interacting pairs were considered as negative samples. Table 4 gives the numbers of positive and negative pair in each cell line.

**Table 4:** Number of enhancer–promoter interactions, enhancers and promoters in each cell line

Cell line	#true EPIs	#all EPIs	#Enhancers	#Promoters
IMR90	1254	2504	108,996	5253
NHEK	1291	2571	144,302	5254
HUVEC	1524	3044	65,358	8180
HeLa-S3	1740	3480	102,460	7794
K562	1977	3952	82,806	8196
GM12878	2113	4226	100,036	8453
HMEC	1342	2684	155,328	5267

^#^Denotes the number

For each positive/negative sample, sequences of enhancers are extended or cut to 3 kb flanking regions on location center of enhancers, and promoter are extended or cut to 2 kb flanking regions on location center of promoters. One-hot coding format of enhancer and promoter sequence is used as input data of model.

We examined the overlapping number of positive EPIs between any two cell lines. For any two positive EPI pairs from any two cell lines, if the position of the two enhancers and the position of the two promoters both same, the two EPI pairs are considered to overlap. By comparison, positive samples of different cell lines have very little overlap (Additional file 1: Table S9).

For comparison with RIPPLE based on epigenetic data, we used data sets from the Roadmap project for the six cell lines. Because we want to make prediction across cell lines, we downloaded the peak files of 14 data sets that are measured in all six cell lines. These data sets include CTCF, POLR2A, H2AZ, H3K27ac, H3K27me3, H3K36me3, H3K4me1, H3K4me2, H3K4me3, H3K79me2, H3K9ac, H3K9me3, H4K20me1 and DNase-seq. An enhancer or promoter sample is represented as a binary vector in which each dimension corresponds to one of the epigenetic data sets. The feature vectors of enhancer and promoter are concatenated to represent each EPI pair. In addition, we also used other two features (i.e., the Pearson’s correlation of the 14 signals associated with the enhancer–promoter pair, and the mRNA level of the gene associated with the promoter) to represent each EPI pair.

### SEPT

Domain adaptation [28] is defined as that source domain and target domain share features and categories, but feature distribution is different. The source domain samples with rich information are used to improve the performance of the model in target domain prediction. The source domain has abundant supervised labeling information, and the target domain has no or few labels. Because SEPT used the idea of domain adaptation, we therefore describe the input data in domain adaptation terms. As focusing on the task of EPIs prediction across cell lines, we assume that there is no labeled training data available in test cell line, only the locations of enhancers and promoters are provided. So, we can utilize the abundant supervised labeling information of other cell lines (called source domain) to improve the performance of EPIs prediction in new cell lines without labeled data (called target domain).

**Fig. 4:**
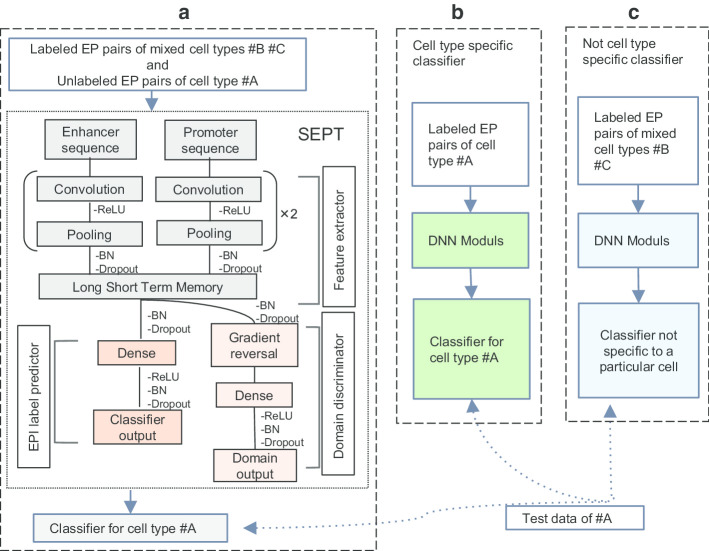
Deep neural network-based methods for predicting EPIs. **a** SEPT architecture. SEPT uses the feature extractor to learn EPIs-related features from the mixed cell line data, meanwhile the domain discriminator with the transfer learning mechanism is adopted to remove the cell-line-specific features and retain the features independent of cell line. EPI label predictor identifies the EPIs in new cell line based on the learned features. **b** Previous model trains a model on specific cell line data, in which the training and test data are both from same cell line. **c** Mixed cell line data are used to train a general model for predicting EPIs in a new cell line

An overview of SEPT is shown in Fig. 4a. For predicting the EPIs in cell line #A, unlike the existing two methods which extract the specific features (in Fig. 4b) or shared features (in Fig. 4c) of cell lines, SEPT uses the rich information of cell lines #B and #C to extract the features that are relevant to the EPIs in cell line #A by using the transfer learning (TL). As shown in Fig. 4a, SEPT mainly includes feature extractor, domain discriminator and EPI predictor. SEPT simultaneously trains two classifiers of the main label classifier and the domain discriminator. These two classifiers share feature extractor layers. It is worth mentioning that we used GRL to design the domain discriminator. GRL reverses the direction of the gradient during the back propagation, but it does nothing when forward propagation. The mixed data of labeled EP pairs of cell line #B and cell line #C are used as the source domain data, and the data of unlabeled EP pairs of cell line #A are used as the target domain data. Each training sample has a domain label, with 0 indicating that the sample belongs to the source domain, and 1 indicating that the sample belongs to the target domain. Each mini-batch training data contains an equal number of samples from both source and target domains. In each training iteration, the parameters in feature extractor layers and EPI label predictor layers are updated on the source domain data, while the parameters in feature extractor layers and domain discriminator layers are updated on both the source and target domains data. In other words, in each training iteration, the feature extractor layers learn the features related to EPI from the samples of cell line #B and #C during the first back propagation, while during the second back propagation, the features learned in the feature extractor layers cannot distinguish which cell line the samples come from due to the GRL. With the training going on, SEPT gradually learns the features which are related to EPI and not related to cell lines.

Feature extractor consists of two convolution layers, two max-pooling layers, two dropout layers, and one recurrent long short-term memory (LSTM) layer. Domain discriminator consists of the GRL, one dense layer, one dropout layer, and the output. EPIs predictor consists of one dense layer, one dropout layer, and the output. Since informative features may differ between enhancers and promoters, we use two convolution layers, rectified linear unit (ReLU) and max-pooling layers for enhancers and promoters, respectively. Thus, the inputs are two one-hot matrixes to represent enhancer and promoter sequence, respectively. Because large number of kernels can sufficiently extract the features, and motif features of DNA sequences are short than 40 bp, so each convolution layer consists of 300 ‘kernels’ with length 40. Max-pooling layer reduces the output dimension with pool length 20, stride 20. The outputs of the two branches are concatenated into one tensor, which is input to the dropout layer with dropout rates of 0.25. The dropout layer randomly selects partial input data to next layer to avoid overfitting. The recurrent LSTM layer is used to extract feature combinations of the two branches, and the output dimension of LSTM is 100. For domain discriminator, the output of LSTM layer feeds into GRL, and a dense layer maps the learned distributed features to the domain label space. It contains 50 units with ReLU activations. The output feeds into a sigmoid unit to predict the domain probability after dropout layer with dropout rates of 0.5. For EPI predictor, the output of LSTM layer feeds into dense layer, which further maps the learned distributed features to the sample label space. It contains 100 units with ReLU activations. The output feeds into a sigmoid unit to predict the probability after dropout layer with dropout rates of 0.5.

#### SEPT model training

We trained SEPT for 80 epochs with mini-batches of 64 samples by back-propagation. In the training phase, source domain data were used to train the feature extractor and the EPI predictor, and both source and target domain data were used to train the feature extractor and the domain discriminator. SEPT seeks to minimize the loss of EPIs label and domain discriminator. Binary cross-entropy loss function for both EPIs label predictor and domain discriminator is used, which is minimized by stochastic gradient descent (SGD) with initialized learning rate initialized equals 0.001. In view of the two optimization objectives, SEPT learns a discriminative representation for EPI prediction and indistinguishable representation for domain prediction. The objective function of the SEPT is defined as follows:

1 $$\begin{aligned} &{\text{E}}\left( {\theta _{f} ,\theta _{y} ,\theta _{d} } \right) \\ & = \sum\limits_{{i = 1}}^{N} {L_{y} } \left( {G_{y} \left( {G_{f} \left( {x_{i} ;\theta _{f} } \right);\theta _{y} } \right),y_{i} } \right) \\ & \quad - \lambda \sum\limits_{{i = 1}}^{M} {L_{d} } \left( {G_{d} \left( {G_{f} \left( {x_{i} ;\theta _{f} } \right);\theta _{d} } \right),d_{i} } \right) \\ & = \sum\limits_{{i = 1}}^{N} {L_{y}^{i} } \left( {\theta _{f} ,\theta _{y} } \right) - \lambda \sum\limits_{{i = 1}}^{M} {L_{d}^{i} } \left( {\theta _{f} ,\theta _{d} } \right) \\ \end{aligned}$$

Here, ${\mathrm{L}}_{\mathrm{y}}$ is the loss of EPIs label predictor, ${\mathrm{L}}_{\mathrm{d}}$ is the loss of domain discriminator, ${\mathrm{G}}_{\mathrm{f}}$ is a mapping that maps the input x to a feature vector,${\mathrm{G}}_{\mathrm{y}}$ is a mapping that maps the feature vector to the EPIs label, ${\mathrm{G}}_{\mathrm{d}}$ is a mapping that maps the feature vector to the domain label, ${\mathrm{x}}_{\mathrm{i}}$ is the i-th sample, ${\uptheta }_{\mathrm{f}}$ is the parameters of mapping ${\mathrm{G}}_{\mathrm{f}}$, ${\uptheta }_{\mathrm{y}}$ is the parameters of mapping ${\mathrm{G}}_{\mathrm{y}}$, ${\uptheta }_{\mathrm{d}}$ is the parameters of mapping ${\mathrm{G}}_{\mathrm{d}}$, ${\mathrm{y}}_{\mathrm{i}}$ is the EPI label of the i-th sample, ${\mathrm{d}}_{\mathrm{i}}$ is the domain label of the i-th sample, N is the number of labeled EPI training samples, M is the number of unlabeled EPI training samples but they have the domain labels, and $\uplambda$ is a constant that controls the tradeoff between two objectives.

It is a problem of minimax optimization, that is, we attempts to seek a saddle point of the functional $\mathrm{E}\left({\uptheta }_{\mathrm{f}},{\uptheta }_{\mathrm{y}},{\uptheta }_{\mathrm{d}}\right)$ that is delivered by parameters ${\widehat{\uptheta }}_{\mathrm{f}}$,${\widehat{\uptheta }}_{\mathrm{y}}$, and ${\widehat{\uptheta }}_{\mathrm{d}}.$ At the saddle point, the loss of EPI label predictor and domain discriminator is minimized. The loss of EPI label predictor is minimized by the feature mapping parameter ${\uptheta }_{\mathrm{f}}$, while the loss of domain discriminator is maximized by ${\uptheta }_{\mathrm{f}}$ on account of the GRL that changes the sign of the gradient during the back-propagation. In the end, SEPT learns the features that are discriminative and domain-invariant. The features learned from the cell lines with label information (source domain) are effective for the new cell lines (target domain).

The training procedure of SEPT can be described as follows: (i) Randomly separating the dataset of target domain into approximate equal two parts, one as the training data in which each sample has domain label but no EPI label, and the other as the testing data. (ii) Mixing the data from other six cell lines and randomly shuffling the data. The mixed data are used as source domain dataset in which each sample has both domain and EPIs labels. (iii) Training SEPT with the source domain data and target domain data. (iv) Evaluating the performance of SEPT with the test data in target domain.

All the experiments are based on Python by using the scikit-learn machine learning library [48] and Keras framework (https://keras.io/) with Tensorflow as back-end [49].

### Evaluation metrics

We adopted the metrics of Accuracy, Precision, Recall, F1-score, AUC and AUPR to assess the performance of SEPT. These metrics are defined respectively as the following [50–53].

2 $$\mathrm{Accuracy}=\frac{\mathrm{TP}+\mathrm{TN}}{\mathrm{TP}+\mathrm{TN}+\mathrm{FN}+\mathrm{FP}}$$

3 $$\mathrm{Precision}=\frac{\mathrm{TP}}{\mathrm{TP}+\mathrm{FP}}$$

4 $$\mathrm{Recall}=\frac{\mathrm{TP}}{\mathrm{TP}+\mathrm{FN}}$$

5 $$\mathrm{F}1=\frac{2\times \mathrm{Precision}\times \mathrm{Recall}}{\mathrm{Precision}+\mathrm{Recall}}$$

where *TP* is the number of correctly predicted EPIs, *TN* is the number of correctly predicted non-EPIs, *FP* is the number of incorrectly predicted EPIs and *FN* is the number of incorrectly predicted non-EPIs. AUC is the area under the receiver operating characteristic (ROC) curve which is the plot of the true-positive rate (i.e., sensitivity) as a function of false-positive rate (i.e., 1-specificity) based on various thresholds. AUPR is the area under the precision-recall curve which is the plot of the precision as a function of recall based on various thresholds.

## Supplementary information

**Additional file 1**. Supplementary materials.

## Data Availability

The related source codes and datasets are available at https://github.com/NWPU-903PR/SEPT.

## Abbreviations

EPIs: Enhancer–promoter interactions
AUC: Area under the receiver operating curves
3D: Three-dimensional
3C: Chromosome conformation capture-based approach
CNN: Convolutional neural network
GWAS: Genome-wide association studies
TL: Transfer learning
GRL: Gradient reversal layer
LSTM: Long short-term memory
ReLU: Rectified linear unit
SGD: Stochastic gradient descent
AUPR: Area under the precision-recall curves
lncRNA: Long non-coding RNA
miRNA: Micro RNA
