# Supplementary material for: Prediction of enhancer–promoter interactions using the cross-cell type information and domain adversarial neural network
Source: BMC Bioinformatics. 2020 Nov 7;21:507. doi: 10.1186/s12859-020-03844-4 (PMC7648314; doi:10.1186/s12859-020-03844-4)
Supplement: Supplementary file 1 — Additional file 1. Supplementary materials. [file 12859_2020_3844_MOESM1_ESM.docx]

**Supplementary Materials for**

Prediction of enhancer-promoter interactions using the cross-cell information and domain adversarial neural network

Fang Jing^1^, Shao-Wu Zhang^1,*^ and Shihua Zhang^2,3,4*^

^1^Key Laboratory of Information Fusion Technology of Ministry of Education, School of Automation, Northwestern Polytechnical University, Xi’an 710072, China

^2^NCMIS, CEMS, RCSDS, Academy of Mathematics and Systems Science, Chinese Academy of Sciences, Beijing 100190, China

^3^School of Mathematical Sciences, University of Chinese Academy of Sciences, Beijing 100049, China

^4^Center for Excellence in Animal Evolution and Genetics, Chinese Academy of Sciences, Kunming 650223, China

*To whom correspondence should be addressed.

Table S1. Average AUPR of SEPT, SPEID, LS-SVM and RIPPLE on seven test cell lines.

| **Test cell line** | **GM12878** | **HUVEC** | **HeLa-S3** | **IMR90** | **K562** | **NHEK** | **HMEC** |
| --- | --- | --- | --- | --- | --- | --- | --- |
| **SEPT** | 0.77 | 0.83 | 0.82 | 0.82 | 0.78 | 0.81 | 0.82 |
| **SPEID** | 0.62 | 0.65 | 0.60 | 0.67 | 0.58 | 0.62 | 0.65 |
| **LS-SVM** | 0.60 | 0.65 | 0.63 | 0.63 | 0.60 | 0.65 | 0.63 |
| **RIPPLE** | 0.68 | 0.62 | 0.61 | 0.64 | 0.63 | 0.62 |  |

Table S2. Average F1-score of SEPT, SPEID, LS-SVM and RIPPLE on seven test cell lines.

| **Test cell line** | **GM12878** | **HUVEC** | **HeLa-S3** | **IMR90** | **K562** | **NHEK** | **HMEC** |
| --- | --- | --- | --- | --- | --- | --- | --- |
| **SEPT** | 0.62 | 0.69 | 0.65 | 0.71 | 0.66 | 0.70 | 0.67 |
| **SPEID** | 0.42 | 0.49 | 0.42 | 0.57 | 0.40 | 0.51 | 0.53 |
| **LS-SVM** | 0.51 | 0.58 | 0.55 | 0.54 | 0.50 | 0.56 | 0.54 |
| **RIPPLE** | 0.59 | 0.46 | 0.49 | 0.62 | 0.59 | 0.45 |  |

Table S3. The domain adversarial models.

| **Model** | **Hidden layers** | | | **Hyper-parameters** |
| --- | --- | --- | --- | --- |
|  | Feature learning part | Domain adversarial learning part | Classifier learning part | Number of kernels (32,100,300)  Learning Rate (1e-03,1e-05)  Reversal Rate (0.5, 1) |
| **SEPT** | 2×{ CNN, Maxpooling },  1 bidirectional LSTM; | 1 Gradient reversal,  1 Dense; | 1 Dense layer |  |
| **BASE+LSTM** | 1 CNN, 1 Maxpooling,  1 bidirectional LSTM; | 1 Gradient reversal,  1 Dense; | 1 Dense layer |  |
| **BASE** | 1 CNN, 1 Maxpooling; | 1 Gradient reversal,  1 Dense; | 1 Dense layer |  |
| **BASE+FC** | 1 CNN, 1 Maxpooling,  1 Dense; | 1 Gradient reversal,  1 Dense; | 1 Dense layer | Number of kernels (32,100,300)  Learning Rate (1e-03,1e-05)  Reversal Rate (0.5, 1)  Number of Dense units (20,200) |

* The explored model architectures, hidden layers and hyper-parameters are shown in columns 1, 2 and 3, respectively. 2×{layer} means a layer repeated two times, 1 CNN means a CNN layer, 1 bidirectional LSTM means a bidirectional LSTM layer, 1 Dense means a Dense layer. The grid search strategy is used to optimize the hyperparameters.

Table S4. Average AUPR values of SEPT and SEP by training model on one cell line data and test on another cell line data in running 10 times.

| **Model** | **Test**  **Train** | **GM12878** | **HMEC** | **HUVEC** | **HeLa-S3** | **IMR90** | **K562** | **NHEK** |
| --- | --- | --- | --- | --- | --- | --- | --- | --- |
| **SEPT** | GM12878 | * | 0.70 | 0.72 | 0.72 | 0.72 | 0.67 | 0.70 |
|  | HMEC | 0.62 | * | 0.55 | 0.66 | 0.72 | 0.59 | 0.66 |
|  | HUVEC | 0.65 | 0.68 | * | 0.69 | 0.69 | 0.62 | 0.70 |
|  | HeLa-S3 | 0.65 | 0.68 | 0.73 | * | 0.71 | 0.68 | 0.73 |
|  | IMR90 | 0.61 | 0.70 | 0.65 | 0.62 | * | 0.57 | 0.68 |
|  | K562 | 0.68 | 0.68 | 0.69 | 0.72 | 0.69 | * | 0.72 |
|  | NHEK | 0.66 | 0.69 | 0.69 | 0.73 | 0.70 | 0.63 | * |
| **SEP** | GM12878 | * | 0.56 | 0.62 | 0.59 | 0.57 | 0.55 | 0.56 |
|  | HMEC | 0.52 | * | 0.54 | 0.56 | 0.63 | 0.52 | 0.58 |
|  | HUVEC | 0.59 | 0.55 | * | 0.58 | 0.57 | 0.56 | 0.56 |
|  | HeLa-S3 | 0.57 | 0.56 | 0.60 | * | 0.57 | 0.55 | 0.59 |
|  | IMR90 | 0.52 | 0.65 | 0.56 | 0.56 | * | 0.51 | 0.59 |
|  | K562 | 0.57 | 0.54 | 0.54 | 0.55 | 0.53 | * | 0.54 |
|  | NHEK | 0.52 | 0.60 | 0.56 | 0.55 | 0.56 | 0.51 | * |

Table S5. Average accuracy values of SEPT and SEP by training model on one cell line data and test on another cell line data in running 10 times.

| **Model** | **Test**  **Train** | **GM12878** | **HMEC** | **HUVEC** | **HeLa-S3** | **IMR90** | **K562** | **NHEK** |
| --- | --- | --- | --- | --- | --- | --- | --- | --- |
| **SEPT** | GM12878 | * | 0.57 | 0.56 | 0.59 | 0.58 | 0.59 | 0.56 |
|  | HMEC | 0.54 | * | 0.50 | 0.54 | 0.57 | 0.52 | 0.53 |
|  | HUVEC | 0.56 | 0.56 | * | 0.57 | 0.56 | 0.54 | 0.54 |
|  | HeLa-S3 | 0.54 | 0.55 | 0.57 | * | 0.57 | 0.52 | 0.57 |
|  | IMR90 | 0.52 | 0.58 | 0.56 | 0.52 | * | 0.52 | 0.57 |
|  | K562 | 0.60 | 0.54 | 0.57 | 0.54 | 0.58 | * | 0.57 |
|  | NHEK | 0.54 | 0.57 | 0.59 | 0.58 | 0.59 | 0.54 | * |
| **SEP** | GM12878 | * | 0.52 | 0.55 | 0.53 | 0.53 | 0.51 | 0.52 |
|  | HMEC | 0.50 | * | 0.50 | 0.52 | 0.55 | 0.50 | 0.52 |
|  | HUVEC | 0.53 | 0.52 | * | 0.53 | 0.53 | 0.53 | 0.51 |
|  | HeLa-S3 | 0.54 | 0.52 | 0.51 | * | 0.53 | 0.51 | 0.52 |
|  | IMR90 | 0.51 | 0.53 | 0.51 | 0.52 | * | 0.51 | 0.52 |
|  | K562 | 0.52 | 0.51 | 0.51 | 0.51 | 0.50 | * | 0.51 |
|  | NHEK | 0.51 | 0.52 | 0.51 | 0.51 | 0.52 | 0.49 | * |

Table S6. Average AUPR, F1-score and accuracy values of SEPT and SEP by training on the six cell lines data and test on one other cell line data in running 10 times.

| **Test cell line** | **Cell line(s) of source domain** | **Model** | **AUPR** | **F1-score** | **Accuracy** |
| --- | --- | --- | --- | --- | --- |
| **GM12878** | HMEC, HUVEC, HeLa-S3, IMR90, K562, NHEK | SEP | 0.64 | 0.44 | 0.56 |
|  |  | SEPT | 0.77 | 0.62 | 0.66 |
| **HMEC** | GM12878, HUVEC, HeLa-S3, IMR90, K562, NHEK | SEP | 0.70 | 0.43 | 0.57 |
|  |  | SEPT | 0.82 | 0.67 | 0.71 |
| **HUVEC** | GM12878, HMEC, HeLa-S3, IMR90, K562, NHEK | SEP | 0.69 | 0.50 | 0.56 |
|  |  | SEPT | 0.83 | 0.69 | 0.72 |
| **HeLa-S3** | GM12878, HMEC, HUVEC, IMR90, K562, NHEK | SEP | 0.70 | 0.47 | 0.60 |
|  |  | SEPT | 0.82 | 0.65 | 0.68 |
| **IMR90** | GM12878, HMEC, HUVEC, HeLa-S3, K562, NHEK | SEP | 0.72 | 0.48 | 0.61 |
|  |  | SEPT | 0.82 | 0.71 | 0.73 |
| **K562** | GM12878, HMEC, HUVEC, HeLa-S3, IMR90, NHEK | SEP | 0.65 | 0.40 | 0.56 |
|  |  | SEPT | 0.78 | 0.66 | 0.64 |
| **NHEK** | GM12878, HMEC, HUVEC, HeLa-S3, IMR90, K562 | SEP | 0.70 | 0.54 | 0.61 |
|  |  | SEPT | 0.81 | 0.70 | 0.71 |

Table S7. Top 5 potentially important TFs identified by the first convolution layer of SEPT and SEP.

| **Cell line** | **Element** | **methods** | **Potentially important TFs involved in EPIs** |
| --- | --- | --- | --- |
| K562 | Promoter | In both SEPT and SEP | ZNF563, RXRA, ZNF554, SP3, THAP1 |
|  |  | Only in SEPT | ZBTB6, EBF1, SALL4, ZNF320, **MAFB** |
|  | Enhancer | In both SEPT and SEP | T, ZNF563, RARG, MAF, LYL1 |
|  |  | Only in SEPT | TCF4, **NR3C1**, ETV4, FOXM1, CUX1 |
| GM12878 | Promoter | In both SEPT and SEP | ZNF563, THAP1, RXRA, OSR2, **ZNF341** |
|  |  | Only in SEPT | TEAD4, ELF1, GABPA, ELF3, MYOD1 |
|  | Enhancer | In both SEPT and SEP | T, MAF, LYL1, NFIA, ZNF563 |
|  |  | Only in SEPT | NR2F2, NKX3-1, NR1I3, ETV4, **NR4A1** |
| HeLa-S3 | Promoter | In both SEPT and SEP | THAP1, SP3, ZNF563, PATZ1, RXRA |
|  |  | Only in SEPT | NR1I3, **BATF3**, ZIC3, ZEB1, CTCFL |
|  | Enhancer | In both SEPT and SEP | NR1D1, ZNF563, MAF, GATA2, NKX6-1 |
|  |  | Only in SEPT | ZN547, SOX9, NFIA, **FOXK1**, RBPJ |
| HUVEC | Promoter | In both SEPT and SEP | THAP1, ZNF563, SP3, RXRA, NR1D1 |
|  |  | Only in SEPT | TEAD4, ZBTB6, PGR, ZNF354A, ELF2 |
|  | Enhancer | In both SEPT and SEP | MAF, ZNF563, T, **ZNF335**, ZNF214 |
|  |  | Only in SEPT | ESR2, NKX3-1, FEZF1, SMAD4, SMAD2 |
| IMR90 | Promoter | In both SEPT and SEP | THAP1, RXRA, SP2, ZNF563, SP3 |
|  |  | Only in SEPT | NKX2-5, RBPJ, TEAD4, TCF4, NR2F2 |
|  | Enhancer | In both SEPT and SEP | MAF, ZNF563, **OLIG2**, T, RXRA |
|  |  | Only in SEPT | TFAP2A, ZNF85, NR1I3, NKX3-1, SOX9 |
| NHEK | Promoter | In both SEPT and SEP | THAP1, SP2, ZNF563, SP3, PATZ1 |
|  |  | Only in SEPT | **TFAP2C**, RBPJ, ETV4, EBF1, MYOD1 |
|  | Enhancer | In both SEPT and SEP | ZNF563, NR1H3, CLOCK, NR1D1, GATA1 |
|  |  | Only in SEPT | SMAD2, SMAD4, ETV4, FEZF1, FOXM1 |
| HMEC | Promoter | In both SEPT and SEP | THAP1, ZNF563, SP3, PATZ1, RXRA |
|  |  | Only in SEPT | NFYC, RBPJ, ZBTB6, SOX10, EHF |
|  | Enhancer | In both SEPT and SEP | NR1H3, ZNF563, RXRA, MAFF, THRA |
|  |  | Only in SEPT | ASCL1, **ZNF317**, ZEB1, TFDP1, REL |

Table S8. Potentially important TFs identified by both SEPT and SPEID.

| **Cell line** | **Element** | **Potentially important TFs involved in SEPT and SPEID** |
| --- | --- | --- |
| K562 | Promoter | ZIC1, SP3, VDR, TFDP1, IRF8, CLOCK, KLF6 |
|  | Enhancer | E2F6, FOXK1, TCF7L1, E2F3 |
| GM12878 | Promoter | SP3, ELF2, CLOCK, EHF |
|  | Enhancer | FOXK1, FOXO1, SREBF2, IRF4, SREBF1 |
| HeLa-S3 | Promoter | SP2, SP3, TFAP2B, CLOCK |
|  | Enhancer | FOXK1, FOXO1, EHF |
| HUVEC | Promoter | THAP1, SP4, IRF1, SP2, SP3, ELF2 |
|  | Enhancer | CLOCK, KLF15, TFDP1, TCF7L1, IRF4 |
| IMR90 | Promoter | CLOCK, KLF6, THAP1, SP2, SP3, ELF2, VDR |
|  | Enhancer | CLOCK, VDR, KLF15 |
| NHEK | Promoter | CLOCK, MZF1, THAP1, SP2, SP3, ELF2 |
|  | Enhancer | MZF1, CLOCK, VDR |

* Only the top 50 TFs of SPEID were used to compare.

Table S9. Overlap number of EPIs between any two cell lines.

| **Cell line** | **IMR90** | **NHEK** | **HUVEC** | **HeLa-S3** | **K562** | **GM12878** | **HMEC** |
| --- | --- | --- | --- | --- | --- | --- | --- |
| **#EPIs** | 1254 | 1291 | 1524 | 1740 | 1977 | 2113 | 1342 |
| IMR90 | * | 11 | 0 | 0 | 0 | 0 | 19 |
| NHEK | 11 | * | 0 | 0 | 0 | 0 | 22 |
| HUVEC | 0 | 0 | * | 0 | 0 | 0 | 0 |
| HeLa-S3 | 0 | 0 | 0 | * | 0 | 0 | 0 |
| K562 | 0 | 0 | 0 | 0 | * | 0 | 0 |
| GM12878 | 0 | 0 | 0 | 0 | 0 | * | 0 |
| HMEC | 19 | 22 | 0 | 0 | 0 | 0 | * |


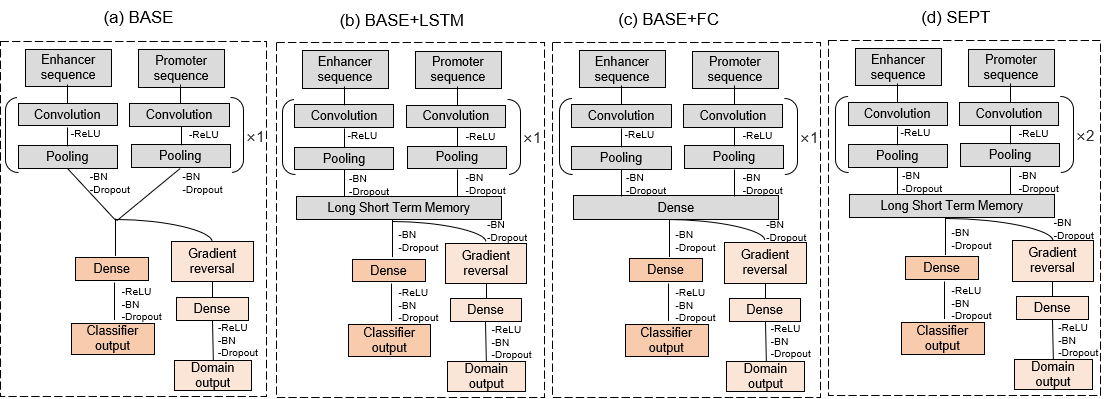


Figure S1. Computational network architectures in feature learning phase. The first network architecture (namely BASE) includes only one convolutional layer in feature learning phase. The second network architecture (namely BASE+LSTM) includes one convolutional layer and one LSTM layer in feature learning phase. The third network architecture (namely BASE+FC) include one convolutional layer and one full connection layer in feature learning phase. SEPT includes two convolutional layers and one LSTM layer in feature learningphase.
